# Peer review of GPT-4 technical report and systems card

**DOI:** 10.1371/journal.pdig.0000417

**Published:** 2024-01-18

**Authors:** Jack Gallifant, Amelia Fiske, Yulia A. Levites Strekalova, Juan S. Osorio-Valencia, Rachael Parke, Rogers Mwavu, Nicole Martinez, Judy Wawira Gichoya, Marzyeh Ghassemi, Dina Demner-Fushman, Liam G. McCoy, Leo Anthony Celi, Robin Pierce

**Affiliations:** 1 Department of Critical Care, Guy’s & St Thomas’ NHS Trust, London, United Kingdom; 2 Massachusetts Institute of Technology, Laboratory for Computational Physiology, Cambridge, Massachusetts, United States of America; 3 Institute of History and Ethics in Medicine, Department of Clinical Medicine, TUM School of Medicine and Health, Technical University of Munich, Munich, Germany; 4 Department of Health Services Research, Management, and Policy, College of Public Health and Health Professions, University of Florida, Gainesville, Florida, United States of America; 5 A.I. and Innovation Committee, Colombian Radiology Association, Medellin, Colombia; 6 ScienteLab, Bogota, Colombia; 7 Be4tech, Medellin, Colombia; 8 Cardiothoracic and Vascular Intensive Care Unit, Auckland City Hospital, Auckland, New Zealand; 9 School of Nursing, The University of Auckland, Auckland, New Zealand; 10 Faculty of Computing and Informatics, Mbarara University of Science and Technology, Mbarara, Uganda; 11 Center for Biomedical Ethics, Stanford University, Stanford, California, United States of America; 12 Department of Radiology, Emory University School of Medicine, Atlanta, Georgia, United States of America; 13 Massachusetts Institute of Technology, Electrical Engineering and Computer Science (EECS), Cambridge, Massachusetts, United States of America; 14 National Library of Medicine, NIH, HHS, Bethesda, Maryland, United States of America; 15 Faculty of Medicine and Dentistry, University of Alberta, Edmonton, Alberta, Canada; 16 Division of Pulmonary, Critical Care, and Sleep Medicine, Beth Israel Deaconess Medical Center, Boston, Massachusetts, United States of America; 17 Department of Biostatistics, Harvard T.H. Chan School of Public Health, Boston, Massachusetts, United States of America; 18 The Law School, Faculty of Humanities, Arts, and Social Sciences, University of Exeter, Exeter, United Kingdom; Mayo Clinic, Arizona, UNITED STATES

## Abstract

The study provides a comprehensive review of OpenAI’s Generative Pre-trained Transformer 4 (GPT-4) technical report, with an emphasis on applications in high-risk settings like healthcare. A diverse team, including experts in artificial intelligence (AI), natural language processing, public health, law, policy, social science, healthcare research, and bioethics, analyzed the report against established peer review guidelines. The GPT-4 report shows a significant commitment to transparent AI research, particularly in creating a systems card for risk assessment and mitigation. However, it reveals limitations such as restricted access to training data, inadequate confidence and uncertainty estimations, and concerns over privacy and intellectual property rights. Key strengths identified include the considerable time and economic investment in transparent AI research and the creation of a comprehensive systems card. On the other hand, the lack of clarity in training processes and data raises concerns about encoded biases and interests in GPT-4. The report also lacks confidence and uncertainty estimations, crucial in high-risk areas like healthcare, and fails to address potential privacy and intellectual property issues. Furthermore, this study emphasizes the need for diverse, global involvement in developing and evaluating large language models (LLMs) to ensure broad societal benefits and mitigate risks. The paper presents recommendations such as improving data transparency, developing accountability frameworks, establishing confidence standards for LLM outputs in high-risk settings, and enhancing industry research review processes. It concludes that while GPT-4’s report is a step towards open discussions on LLMs, more extensive interdisciplinary reviews are essential for addressing bias, harm, and risk concerns, especially in high-risk domains. The review aims to expand the understanding of LLMs in general and highlights the need for new reflection forms on how LLMs are reviewed, the data required for effective evaluation, and addressing critical issues like bias and risk.

## Introduction

### New opportunities and potential benefits of large language models (LLMs)

The natural language processing (NLP) field is advancing at an extraordinary pace in both academic performance and integration with the broader public [1]. Generative Pre-trained Transformer 4 (GPT-4) marks the latest in this field’s series of significant leaps [2,3]. OpenAI has released an extensive technical report (TR) in the style of a formal academic paper demonstrating exceptional results [2]. For example, GPT-4 scored in the top 10% of test takers across numerous bodies of knowledge in simulated examinations [2].

New horizons opened by LLMs have been extensively discussed, already with significant applications in the biomedical and pharmaceutical domains, such as protein design and drug discovery [4,5]. Many also envision LLMs will substantially impact beyond "AI for health sciences" and eventually transfer into "AI for healthcare practice." Namely, their potential use in reviewing clinical documentation, answering questions about electronic health record (EHR) data, augmenting clinical procedures, and helping radiologists to produce grounded radiology reports are all potential applications [6–8].

### AI has greatly benefitted from and necessitates transparency

Like many other computer science publications, the OpenAI report is publicly available at arXiv. However, the capabilities and scale of GPT-4 have resulted in novel safety challenges that must be safeguarded against [9]. Like any machine learning algorithm, GPT-4 may have inherent biases that reflect biases in training data. In addition, the safety risks listed by the authors include the following:

Hallucinations, Harmful content, Harms of representation, allocation, and quality of service, Disinformation and influence operations, Proliferation of conventional and unconventional weapons, Privacy, Cybersecurity, Potential for risky emergent behaviors, Interactions with Other Systems, Economic impacts, Acceleration, and Overreliance.

Traditionally, the artificial intelligence (AI) field has greatly benefitted from the Open-first approach, from ImageNet to MNIST and MIMIC. Therefore, for GPT-4 to go against this grain, at present, where the data used for training and the necessary compute for training remain a trade secret, raises old concerns. Initial hesitance in releasing LLMs due to potential societal impact was common, yet it needs to be clarified if these obstacles have been overcome or what has changed in this period [10,11]. Especially when we review the model for bias, we are now speculative—as many researchers will not have access to the resources and datasets needed to train their own LLMs. Without systems and standards safeguarding against potential risks, self-audit can produce selective sharing and expose humans and patients to risks.

In the context of GPT-4, the OpenAI report is highly selective in disclosing foundational elements, such as training data, necessary to scrutinize critical aspects, including representativeness, robustness, and quality of the datasets. To ensure fairness and equity, it is essential to identify and address such biases and ensure that the model is trained on diverse and representative data. This self-selection is further demonstrated by the number of redactions released from the final draft, including areas such as toxic language. While this also indicates that the TR was meant as less of a rigorous technical evaluation and more of a demonstration of current performance, it highlights the potential for omitting areas deemed damaging. Indeed, review of the LaTex source code on initial drafts of the paper has highlighted the internal dynamics of this process, such as significant reduction of sections on “Toxic Content.”

The rising number of applications that have links to GPT-4 across education [12,13], finance [14,15], government [16], and healthcare [17] is exacerbating concerns. This is compounded by the proliferation of startups worldwide utilizing the technology and the growing visibility of the AI economy to consumers [18,19]. The TR proactively addresses this omission by referencing the competitive landscape and notes possible further disclosure later. While this may justify some of the nondisclosure, the gravity of potential harms in the interim that could result from lack of access to this information justifies an expectation of greater efforts toward more adequate stopgap measures.

### This study reviews the GPT-4 report and summarises critical strengths and weaknesses

The importance of technical details in foundational models cannot be recognized, particularly when applying to high-risk settings. OpenAI states they could release such pending a legal process. Herein, we aim to set a blueprint for future technical descriptions of LLMs that maximize benefits and mitigate risks while respecting the competitive landscape. We highlight areas of strength in the current report and key areas of omission and set this in the broader context of LLMs. We focus on the implications to the healthcare domain due to our expertise and the importance of the systems evaluation before use in an extremely high-risk application. We frame our review of this highly consequential topic by emphasizing the importance of developing and evaluating appropriate uses for LLMs and mitigating the risks associated with applications [10,20].

This study aims to provide an impartial review of this technology, specifically this TR and its associated systems card. We conducted a peer review of this report using guidelines from previous publications and domain expertise spanning multiple fields, including engineering, computer science, law, and healthcare. Finally, we highlight critical questions raised and summarize a list of recommendations to address before this technology is deployed at large.

## Methods

### Technical report

For ease of reviewing and presentation of results, the document was downloaded from arXiv on March 22, 2023, converted to Microsoft Word in Adobe Acrobat, added line numbers, and converted back to PDF format. No other alterations were made to the published document. The full report with line numbers used can be found in S1 File.

### Reviewer composition

A group with geographical and disciplinary diversity was necessary to deliver the most comprehensive perspective on this technology. Reviewers from across the globe with a wide range of professional training were sought to provide a global perspective on this innovation. The following areas were decided as critical to the success of the review: AI, NLP, public health, law, policy, social science, healthcare research, and bioethics. A full breakdown of the author’s expertise and background can be found in S2 File.

### Review criteria

The authors reviewed the report individually before all feedback was assimilated into a final document. Reviewers were asked to consider review criteria in S3 File based on domain expertise and previous literature; however, reviewers were free to comment on any aspect of the manuscript [21,22]. Responses were then grouped into key categories, and all reviewers reviewed the final responses. No responses were omitted.

## Findings

Feedback from 12 reviewers was included from 6 countries and 4 continents. The review has been split into Strengths and Weaknesses, and Table 1 provides an overview of the significant points.

**Table 1:** Summary of GPT-4 TR review.

Reviewers’ Comment	Explanation
**Strengths**	
Significant time and economic commitment to transparent AI research.	This report offers significant detail and results in the performance of GPT-4. This comes at considerable time and economic expense, which the authors invested despite knowing it will likely not be read by many users.
Creation of a systems card that explores risks and mitigations.	Exploring risks, developing mitigation tools, and associated frameworks are essential to AI deployment. This report has extensive analysis and proposes a framework for model evaluation.
**Limitations**	
Limited access to training data and processes prevents confidence over bias.	A model’s outputs reflect the data they are trained upon; therefore, without clarity of training processes and data, the extent and ways in which GPT-4 contains encoded biases and interests of third-party data sources is unclear.
More confidence and uncertainty estimation are needed, particularly in high-risk settings.	GPT-4 will perform differently on different tasks and spheres of knowledge based on the information it is trained on. However, without providing users with an estimation of confidence, each response will be taken with the same, despite different performances. This is particularly troublesome in high-risk settings, such as healthcare.
Concerns of privacy and IP should be acknowledged.	Data used to train GPT-4 will be used in outputs. However, if GPT-4 has been trained on personal or sensitive information, then this is at risk of being revealed to another user. This is also true for any text that contains details of documents and codes covered by IP rights.
LLMs will require multiple teams’ input during design, evaluation, and oversight.	Biases are complex and require multiple teams to evaluate a model for different pockets of response types. In addition, external input will be required to ensure that a “competitive landscape” does not lead to conflict with data privacy or the perpetuation of biases.
World-changing technology should include values from around the world.	The outputs of this model that are deemed appropriate will reflect the training processes and feedback given. Therefore, there is the potential to lock in the values of the group designing this process. To ensure this technology benefits all groups, a diverse group from around the world should be involved in this process.

AI, artificial intelligence; GPT-4, Generative Pre-trained Transformer 4; IP, intellectual property; LLM, large language model; TR, technical report.

### Strengths of the technical report

#### Significant time and economic commitment to transparent AI research

Evaluating an LLM’s strengths, weaknesses, and dangers before deployment is commendable. The report’s authors extensively test the model on different standardized tests and languages. The results are impressive, and the collection of such developments into one very readable document demonstrates a significant commitment to transparency that is not mandatory or universally applied. The breadth and depth of information provided in this report are commendable and would have required considerable time and resources to develop. This is particularly impressive given that this is likely to be read by a small percentage of the innovation’s users.

The report names several criteria for *model testing*, or evaluating *model output*, and deployment readiness, or potential for harmful applications by model users, with the latter being a hard-to-control yet essential ethical and legal consideration for the model developers. Specific to model testing, the report includes and operationalizes the following criteria (with additional healthcare-specific commentary our own):

- **Hallucinations** can lead to unreliable and misleading output from LLMs. Some of the “hallucinated” content may be nonsensical, but this may also include content with misleading or false information about medical conditions, treatments, or medications, leading to dangerous or ineffective health practices.
- **Harmful content** involves the generation of content that can cause harm to humans, such production of violent content, advice on behaviors detrimental to self or others, or instructions for finding illegal content. In the healthcare context, this could include generating materials promoting self-harm or instructing methods of suicide.
- **Harms of representation** include producing content that perpetuates societal stereotypes, amplifies biases, and promotes some societal norms while silencing others. LLMs can bring about innovation and improved health outcomes, but the GPT-4 TR calls for careful evaluation of model outputs that can contribute to disparities and bias toward minority and marginalized groups. Past work has already demonstrated the potential for natural language models to embed harmful stereotypes, such as agitated Black patients being sent to prison, with equivalent white patients sent to hospital [23].
- **Privacy infringement** includes the collecting and augmentation of publicly or institutionally available personal information, which together can produce protected information about persons. Access to voice and health information on social media further enables LLM models to generate a personal profile violating Health Insurance Portability and Accountability Act (HIPAA) or other privacy regulations.
- **Power-seeking**, or potential for risky emergent behaviors, manifests in models’ ability to define and pursue long-term goals that may go beyond the original intent of the model developers. In health-related contexts, this may include a model originally designed to share information about clinical trials in support of participant recruitment but learns overly persuasive language, thus undermining informed consent.

Similarly, the report discusses criteria for deployment readiness evaluation, including risks for model use for disinformation and operation influence, the proliferation of conventional and unconventional weapons, cybersecurity attacks, interactions with other systems, economic impacts, acceleration, and overreliance. Across all criteria, the report authors relied on human evaluators who assessed the quality and coherence of the generated text using specific examples that can trigger and identify any problematic content output. The report, therefore, contributes to future basic and applied research by helping to articulate the evaluation metrics and providing examples of reporting. Future applications of GPT-4 in local contexts should include the effort to conduct, document, and report their model testing evaluations, potentially enhanced by testing against existing content and databases. Furthermore, implementation of GPT-4 must be considered as part of an ongoing systematic process that considers capacity to address novel privacy risks, maturity of associated infrastructure, and other use challenges discussed in the report.

#### Creation of a systems card that explores risks and mitigations

The limitations section, risk and mitigations, and the system card provide insights into the steps taken to understand and mitigate safety concerns. This is extensive and outnumbers the TR significantly in length. In addition, there is a description of a robust framework for future evaluation of GPT-4 performance, which can serve as guidance for local model implementations.

### Limitations of the technical report

#### Limited access to training data and processes prevent confidence over bias

GPT-4 is stated to be trained on publicly available data, such as the internet and data licensed from third-party providers (lines 63 to 68). The authors report that the second stage involves model fine-tuning using reinforcement learning from human feedback (RLHF). However, further details on the training set are not described due to concerns surrounding safety implications and the “competitive landscape.” The training set’s characteristics are widely acknowledged for contributing significantly to biases introduced during model development and may have significant implications for patient outcomes [11,24]. Further, using RLHF is a helpful tool to increase the appropriateness of responses by a model. However, depending on the demographics and agendas of labelers, specific responses will be favored over another. Details of who was included in the active learning process, including their demographics and background, and instructions given to labelers are essential to understand the logic and values encoded in the model, as well as potential biases in the feedback data. It is clear from both the TR and other OpenAI research that RLHF has a substantial impact on model performance and accuracy, which is at times deleterious [20]. Yet, details of the impact of RLHF on ethical and safety-related outcomes remains broadly qualitative and limited in scope.

The use of data from third parties is common in NLP. However, its use in GPT-4 may have troubling implications given its role in providing facts or opinions on facts and therefore needs further information. First, using third-party providers’ data requires additional explanation on multiple fronts. The authors likely refer to data to prepare for benchmark testing (lines 211 and 1,442). However, this provides inadequate information to end users or those whose data are being used. That is, without additional information about which third-party sources are used, it is impossible to know if providers are aware that these data are being shared for this purpose. The consequences of this can be considerable, ranging from the use of illegally obtained data to data derived from questionable sources, possibly under dubious circumstances. Beyond this, it is conceivable that the use of data sourced from third parties may ultimately serve to operate contrary to the interests of that third-party source. It may be the case that providers have a vested interest in LLM learning or generating a particular view of the world that benefits that third party.

#### Poor confidence estimation and reliability should limit the application to specific settings

There is limited exploration of attempts to quantify uncertainty in a response or attempts to provide reasoning behind an answer, for example, a source used to generate a response. While it is common to approximate language understanding with multiple choice exams, it is not clear what downstream task is emulated in these experiments other than potentially revealing problems with standard testing. In addition, model evaluation scenarios should consider real-life scenarios in which the models will be used. A significant use case of LLMs is generating short answers to questions, which is not evaluated in multiple-choice settings.

The limited effort to quantify uncertainty with each output or explicate the underlying mechanisms or sources makes it very difficult for users to evaluate the merits of the sources and to seek verification where sources may raise questions. As a result, applying this model in or regarding high-risk activities, such as healthcare, should be considered seriously before being deployed. The authors acknowledge that the reliability of outputs cannot be fully guaranteed, and, therefore, limiting applications with high-stakes contexts is essential (lines 47 to 53, 427 to 434, and 477 to 479). However, the application of this technology is already being linked to such settings [17,25].

#### Concerns about privacy and intellectual property should be acknowledged

The authors have used several processes to reduce the risk that GPT-4 is used to identify personal data, including monitoring and rejecting requests to perform such a task. The authors also aim to remove personal information from the training dataset where feasible. This is similar to publicly available clinical datasets. However, it must be noted that without an explicit understanding of what data were in the dataset, it is difficult to evaluate the feasibility and success of deidentification. Nevertheless, these control measures are valuable for limiting direct attempts to identify personal data. However, such measures are unlikely to address the reidentification of personal data that becomes increasingly feasible in the context of large amounts of data drawn from public and private sources [26]. These further issues surrounding data privacy prompt questions about what assurances must be provided to users regarding the risk of being reidentified. Notably, the most recent proposal of the EU AI Act specifically requires that providers of “foundation models,” of which generative AI (ChatGPT-4) is a subset, identify and mitigate foreseeable risks to fundamental rights such as privacy and data protection [27]. However, current agreements used to regulate such processes were produced based on the assertion that data would be put into the public domain under a certain level of risk. If this tool substantially changes that risk, it should be considered whether additional consent or regulation should be required [26]. This is particularly true given the potential applications in the healthcare setting where patient data may be entered into prompts that may be used in subsequent model retraining. The significance of the privacy issues in the healthcare setting is arguably greater given not only the sensitivity of the data but also the heightened risk for serious consequences in the potential use and dissemination of sensitive data. Furthermore, ChatGPT-4 is not designed to be HIPAA compliant, which could substantially curtail its use in the healthcare setting [28]. Moreover, GPT-4 may not have the capacity to attain the health data privacy that HIPAA aims to protect without some loss of functional ability due to advances in reidentifiability of deidentified data. Furthermore, because HIPAA applies only to “covered entities” (involved in the provision of healthcare), the reach of HIPAA is likely to be too narrow to provide sufficient privacy protection. When LLMs designed for medical use are used by healthcare institutions and, as covered entities are required to comply with, HIPAA does not address issues of reidentifiability as that may now manifest in the context of models developed using unknown sources of data derived from publicly available data. As a result, even if GPT-4 is HIPAA compliant, it is not altogether certain that this provides the protections envisaged by HIPAA when enacted because of the scale and scope of data collected.

This is also true for using current data collected by institutions with links to LLMs. Consider the amount of text available to Microsoft through Outlook, Word, or Google through Gmail and Google Docs. This may contain sensitive or protected data that could cause personal, collective, or industrial harm. Conversations around the social license to share these data or not should be discussed before these data’s usage, even if it already has occurred. Additionally, there have been recent reports of employees leaking sensitive company data [29].

#### LLMs will require multiple teams’ input during design, evaluation, and oversight

The authors request further research to characterize risks that emerge from LLMs generally and across different language models to guide the development of these models in safer directions. The authors also report working on these types of evaluations, often in collaboration with other research groups, focusing on assessing risky emergent behaviors. However, this is not easy to conduct in practice without disseminating details surrounding the training data, model architecture, and associated training processes. While this information may be shared with these specific groups, whether one group can fully evaluate a model for all these biases must be questioned and, if they should, requires further deliberation. The complexity of biases encoded in algorithms is often far beyond what one group can identify; thus, a citizen science or open science approach may be more beneficial. These models enable a wide range of use cases, and the evaluation of risks must be enabled through openness to be similarly context specific. Several organizations are disseminating tools to detect LLM-generated content for education. The performance of these tools is dismal (likely affected by limited access to the LLM training data and code), with ongoing reports of student cheating based on these models that propose to be a stopgap [30].

In addition, the economic potential of this model and other LLMs again brings up questions of intellectual property, which may act in competition with the safest route to deployment. Whether one organization can be expected to conduct a rigorous evaluation of models for such biases to the standard of an independent regulator is questionable. Further, it is also doubtful if they should be trusted not to use data available to them that would improve model performance but cross the gray areas of data privacy. This is particularly true in a “competitive landscape.” While risk mitigations may reduce certain biases, misuses, or misinformation, these issues must be met with anticipatory planning and governance, as the authors also state [31]. This is particularly true in a high-risk setting such as healthcare where the use of sensitive information may ultimately result in life and death decisions being made. A middle path must therefore be created that permits competition to occur safely, and this will require input from governments and the public alike.

#### World-changing technology should include values from around the world

There was a solid effort in the TR to include professionals with experience in critical areas such as fairness and disinformation, and names of adversarial testers and red teamers are included (lines 1,173 to 1,184 and 2,082 to 2,095). It is also acknowledged that participants were selected based on prior research or experience in these areas, which may introduce bias. The composition of this group is noted to be weighted towards Western countries and those from traditional higher education institutes. The authors recognize that this will shape the values and perceived risks associated with the model; however, it is essential that broader perspectives are sought in future iterations. Values must reflect those from a wide variety of backgrounds, not just those of a particular socioeconomic group or educational training. Further, the impact of this technology could be world-changing, and, thus, worldwide representation should be encouraged. This is necessary to ensure that such a technology’s benefits are beneficial for regions that have traditionally been overlooked or actively exploited by technological development. While experts must be sought to evaluate tools that demand significant expertise, this does not preclude the involvement of wider disciplinary teams, particularly when considering the implications of their deployment.

The report notes that the GPT-4-early version was tested and later trained to withhold harmful advice or information, such as information about harm to self and others or instructions on finding illegal or hateful content. The report also discusses that the model was later corrected to allow harmful yet legal content, such as information about low-cost tobacco products. However, the report is silent on the normative foundations and models used to create an ontology of permissible and nonpermissible information. In effect, the review of the GPT-4 needs to include explainability and transparency around the cultural, ethical, legal, and social values it propagates.

## Discussion

The goal of this open peer review was 2-fold: (1) to critically assess the rigor, transparency, and reproducibility of the GPT-4 technical report and systems card; and (2) to map the transdisciplinary research agenda that can support the ethical and sustainable development of AI innovation in healthcare. Next, we will summarize the findings and then provide suggestions for future research.

This report demonstrates the significant leap forward of GPT-4 and offers a detailed contribution of the risks and limitations of such technology. While key risks are explored, there is a lack of detail in fundamental areas such as data sources, training processes, and details surrounding user privacy. OpenAI describes several critical areas of future research that all developers of LLMs should conduct with implications for the whole industry. Table 2 explores the implications of these omissions and purports future research foci for transdisciplinary research on large language models.

**Table 2:** Implications for future LLM research.

Cross-cutting topic	Research agenda/Recommendation
Ethical and legal implications of LLMs	Improve transparency of data used and clarify the role of third-party data. Incorporate a mechanism that allows for greater scrutiny of sources used. Develop a framework for accountability.
Translation of LLM knowledge to high-risk settings	Design standards of confidence in LLM output before translation into high-risk settings. Develop systematic review infrastructure for post-market surveillance.
Academic and industry research.	Establish processes for review of industry research. Develop novel methods to quantify uncertainty in model outputs.
Societal implications and consequences.	Create transdisciplinary partnerships to evaluate model impacts. Develop international and LMIC collaboration to ensure culturally competent performance.

### Concerns of bias and potential for LLMs to harm

While this technology can potentially revolutionize many fields, there is also concern about the potential for these models to spread misinformation or perpetuate bias at scale. This mirrors broader concerns for AI deployment in healthcare, where biases in electronic health records can be transmitted into downstream model performance. However, GPT-4 also brings substantial new concerns to bear in AI debates, such as the tendency to "hallucinate" (or the possibility to generate nonsensical or irrelevant responses), act in unpredictable ways, or struggle with processing complex and nuanced language, such as sarcasm, irony, or metaphor [32]. Furthermore, as language models become increasingly powerful, there is a growing concern about their potential misuses, such as generating hate speech, propaganda, or deep fakes [11].

A significant concern is that the sources used to generate GPT-4 are primarily refractory to scrutiny, leaving users to rely on an output, the basis for which they cannot verify. In addition, the lack of public versioning or documentation of back-end changes renders it difficult to consistently audit model behavior. GPT-4 must be designed and used responsibly to prevent such unethical practices. While it is often the case that it is difficult to foresee all of the risks of new technologies, in the case of GPT-4, some of the most severe risks are obvious, obviating any excuse for not seeking to mitigate them from the outset. We appreciate that Section 2, GPT-4 Observed Safety Challenges, in the GPT-4 System Card presents the harms identified by a large and somewhat diverse group of testers. We also applaud the measures taken towards safety and discouraging problematic use or outcomes, such as warnings against harmful behaviors contained in prompts (see, e.g., smoking) and disallowance of manifestly harmful prompts (e.g., how to make a bomb). These are valuable controls, but, as the authors acknowledge, the models that sound plausible and make fewer mistakes may be more dangerous due to the increased trust that develops with familiarity.

It must be acknowledged that other LLMs in high-risk settings such as healthcare have demonstrated a greater degree of openness in characterizing their development processes. Healthcare-specific LLMs, including MedPaLM from Google [33] and GatorTRON from researchers at the University of Florida and Nvidia [6], have offered extensive detail regarding their training datasets and evaluation processes. In the case of MedPaLM, the Google researchers further developed and validated a quality assurance task suite (MultiMedQA) upon which performance could be compared across models. While OpenAI and Microsoft have performed research subjecting GPT-4 to post hoc evaluation through the MultiMedQA task set and other specific evaluative benchmarks [20], the closed nature of the model architecture heavily restricts the possibility of effective external audit. While the authors give summary statistics and individual example outputs, no systematic dataset of prompts and responses is available. Particularly given ongoing proposals for the inclusion of GPT-4-related models in point-of-care electronic medical records, openness in this regard is important to ensuring safety and clinical buy-in [34].

### Accountability, responsibility, and liability

GPT-4 raises several legal issues in addition to privacy, data protection, and intellectual property, all of which may arise in the production and deployment of the technology. Once the technology is made available to users, potential legal issues may arise due to its use. Generating output that misinforms regarding a high-risk activity or discourages more effective solutions to an urgent need may cause serious harm to a user who has relied on this information. Depending on the disclaimers that accompany consent to the use of GPT-4, there must be some accountability for the provision of foreseeably misleading information on which a user might rely to their detriment. With a technology of this scope, there are limits to caveat emptor (Let the buyer beware). For example, basic considerations underlying product liability require that a product actually be fit for purpose. Failing that, the seller is liable for the cost of the product and foreseeable damages [35].

In the case of GPT-4, a simple (or complex) disclaimer may be all that is needed to satisfy considerations of legal accountability. However, as an ethical matter, the foreseeable and, indeed, expected reliance of users on GPT-4 outputs would suggest that more is needed in the way of accountability. Because of the pioneering nature of several aspects of the technology, it would be reasonable to expect that such a technology would be accompanied by its own framework for accountability in which, at a minimum, it lays out precautions that the OpenAI has taken, precautions that the user should take, and a clear accounting of both direct and indirect risks that could arise in various contexts, including warnings that the technology should not be used for specific purposes [36]. Such warnings do not prevent undesirable uses, but as with any potentially harmful misuse of a product, the warning should serve to elevate caution and foster understanding of the limits of the technology. In doing so, the manufacturer takes on a degree of accountability that ultimately serves both the user and the manufacturer. Importantly, such a framework does not supplant a need for regulatory regard but rather acts as an important complement that can enhance the kind of trustworthiness that supports innovation. Additionally, issues pertaining to copyright infringement through the use of publicly available data and images may require address for any dignitary, proprietary, and economic harm. Notably, the proposed EU AI Act also takes aim at the risk to copyright interests brought about by ChatGPT and has amended the proposed regulation to require transparency specifically regarding any copyrighted material in the data used to develop the model [37]. Furthermore, energy consideration and impact on climate and environment need to be considered and responsibility allocated in sensible and ethically defensible ways.

In the context of generative AI, a framework for accountability also acknowledges a sense of responsibility for the operation and use of the product [38]. Such a framework could identify appropriate measures taken to mitigate risks at various stages in production. In devising schemes of responsibility, one approach looks to the party who is in the best position to mitigate harm (e.g., by design, development, or explicit use limitations), to assume the responsibility to do so. Thus, data brokers, developers, deployers, and providers may carry some degree of responsibility, and, under the revised EU AI Liability rules, exposure to civil liability cannot be avoided simply by the complexity of locating responsibility. The advent of this high-powered technology may also change how liability plays out where a person voluntarily but foreseeably relies on information to their detriment. While there has been little success in holding the providers of information liable for harm caused by such reliance (except in contexts such as healthcare where the point of providing the information is reliance), the particular configuration of sourced, combined, and integrated data in specific contexts, e.g., educational, healthcare, or transport, may result in a need to revisit responsibility and, eventually, liability. The most recently proposed amendments to the EU AI Liability Directive that effectively introduce a presumption of causality may signal an eventual shift in how proof of liability is viewed in the context of AI systems [39]. This may, in turn, inspire new schemes of responsibility. While GPT-4 may not fall under the Liability Directive, the fact of legislative recognition of the complexity of proving causality in the context of the use of AI devices could raise the specter of innovative interpretations of causality.

Regardless of possible legal configurations of allocation of responsibility, in putting GPT-4 on the market, OpenAI must engage with a level of accountability that reflects the awareness of potential use and misuse and meaningful engagement with how harmful sequelae can be minimized. Accountability and responsibility must be understood to extend beyond that which is currently legally mandated to ensure that the trustworthiness that is so essential to the successful uptake and integration of generative AI is not compromised. The proposed EU AI Act, the first comprehensive attempt at regulation of AI, has identified key considerations that should be noted beyond the EU regardless of jurisdiction. Ensuring appropriate disclosures both for proprietary as well as safety reasons, along with documentation of adequate testing and mitigation measures for foreseable risks, including to the environment, are among the necessary points of reference in developing a responsible approach to the provision and deployment of GPT-4.

### Disruptive technology increases the demand for interdisciplinary reviews

Peer review is critical to the scientific process, ensuring research is rigorously evaluated before publication and safeguarding knowledge before implementation. This is especially important for reports on large AI models like GPT-4, which have the potential to impact a wide range of fields and applications. Given the interdisciplinary nature of AI and its applications, experts from different disciplines must conduct peer reviews for these models.

Firstly, large AI models like GPT-4 have applications in various engineering, data, and computer science fields. Each of these fields has its own set of specialized knowledge and techniques. Experts from each area must be involved in the peer review process to evaluate the model from all relevant perspectives. Experts should also realize that it is their duty to conduct such reviews. Secondly, AI models disrupt technical progress and societal norms, cultures, and processes. Given the potential for these models to impact people’s lives, experts from legal and social studies, ethics, anthropology, sociology, history, knowledge transfer, and health services research must be involved in the peer review process. This will ensure that the ethical and social implications of the model are considered alongside its technical capabilities. Finally, the interdisciplinary peer panel involved in this review will help to ensure that the TR receives a holistic, transdisciplinary assessment to recognize the strengths and identify potential issues with the TR that may have been missed if experts from a single field conducted the evaluation.

### Setting up systems for high-risk applications

The translation gap for AI in healthcare is well acknowledged, and LLMs could fall into the same trap [40–42]. Particularly, if we plan to use closed models in this high-risk setting, even more so if we use models that consistently change and update over time. With the lack of version numbers and constant ongoing changes to the model, it is difficult to evaluate the models or for regulatory bodies to certify them properly. The field of MLOps has proposed several approaches for wider healthcare AI applications [43,44]; however, all will require or greatly benefit from increased transparency. The standardization of data collection and reporting should be foundational to its translation, starting in the development process. It is clear that while no group can fully understand all risks and biases or explore these in one document, there should be a structure that permits their exploration. What must be present is an acknowledgment that a systematic process to detect and improve these problems, even after deployment, must be developed; this is a continuous process. To safeguard against biases in clinical data, humans used in RLHF, or model design, an Open-first approach must be a default position.

### Limitations of this peer review

We recognize that this TR was not produced for formal peer review but to provide insight into a novel technology with world-changing potential. This review recognizes GPT-4’s significant advance in the field and cannot criticize its potential to do significant good. However, its far-reaching implications warrant further discussion of essential areas of concern that involve an industry, not just GPT-4 or OpenAI alone. A diverse group conducted this review with respect to expertise and geography; however, in no uncertain terms is this complete. We aim for this review to be a springboard for further open dialogue on this revolutionary technology. Additionally, we encourage other groups to provide their unique insight to maximize the good this technology can do while safeguarding against its potential dangers.

Further, we seek to emphasize that peer review of a document for publication represents only a small part of what is needed for a thriving, open scientific engagement with such an important advance. Just as all outside of a few select researchers, our group is limited in our degree of access to the GPT-4 model, and we must rely primarily upon the materials produced by OpenAI researchers. Beyond mere expansion of these materials or prepublication peer review, we encourage a culture of openness, which will enable evaluation of LLMs and improvement of this technology by a broad research community with diversity befitting the diversity of GPT-4’s potential applications.

## Conclusions

The GPT-4 TR is a step in the right direction toward more open conversations around the workings of LLM. However, the self-audit of LLMs by the tech industry is not sufficient, and significant questions remain unaddressed. In this paper, we have sought to expand the review of GPT-4 and bring a range of disciplinary expertise to bear on its potential concerns, particularly for high-risk areas. In conducting this review, we also intend to point to the need for new forms of increased reflection on how LLMs are reviewed, by whom, what data reviewers need access to, and how concerns such as bias, harm, or risk are addressed.

## Supporting information

S1 File Technical report of GPT-4 with line numbers. (PDF)

S2 File Reviewer profiles. (DOCX)

S3 File Review criteria and prompts to reviewers. (DOCX)
