# Supplementary material for: Peer review of GPT-4 technical report and systems card
Source: PLOS Digit Health. 2024 Jan 18;3(1):e0000417. doi: 10.1371/journal.pdig.0000417 (PMC10795998; doi:10.1371/journal.pdig.0000417)
Supplement: S2 File — (DOCX) [file pdig.0000417.s002.docx]

## **S2 File- Reviewer Profiles**

**Dr Jack Gallifant** is a postdoctoral researcher at MIT's Laboratory for Computational Physiology, stands at the forefront of interpreting and navigating the intricacies of foundational models in machine learning. His primary focus is unraveling the complexities of mechanistic interpretability, particularly in understanding how biases are stored, propagated, and respond to shifts in data within language models. This critical work aims to enhance the ethical deployment and efficacy of AI in healthcare, aligning with the broader goal of equitable and efficient medical solutions.

He is also a trained physician completing foundation training at Imperial College London NHS Trust and has an MSc in Human and applied physiology from King’s College London. He is an honorary researcher in the Department of Critical Care at Guy’s & St Thomas NHS Trust focusing on predicting patient deterioration and detecting biases in clinical devices and algorithms.

**Dr Amelia Fiske** is a Senior Research Associate at the Institute for the History and Ethics of Medicine at the Technical University of Munich, Germany. She is trained as a cultural anthropologist and has been working in interdisciplinary bioethics settings since 2017. Her work is situated at the intersection of cultural anthropology, science and technology studies, graphic art, and social medicine and bioethics. Prior to the TUM, she received her PhD in cultural anthropology from the University of North Carolina at Chapel Hill (USA), held a postdoctoral position at Kiel University, and conducted extensive field research in Ecuador and Latin America. She has over a decade of experience conducting interdisciplinary qualitative and ethnographic research in two broad arenas: 1) anthropological and critical social science approaches to bioethics, artificial intelligence, and digital and sociotechnical changes in knowledge production; 2) ethnographic attention to issues of socio-ecological justice, experiences of toxicity in the context of extraction, participatory research methods, and graphic arts.

**Dr Yulia A. Levites Strekalova** is Assistant Professor of Health Services Research, Management & Policy and Director of Evaluation and Educational Development at the U.F. Clinical Translational Science Institute. Dr. Strekalova’s research is focused on the scholarship and application of team science to facilitate the integration of research into healthcare practice. Dr. Strekalova’s expertise includes program planning and evaluation, teaming to enhance diverse perspectives in biomedical research and practice, and the implementation of human-human and human-machine teaming interventions to achieve the quadruple aim (i.e., improved population health outcomes, reduced healthcare costs, enhanced patient experience, and improved provider satisfaction). Dr. Strekalova is also Director of Evaluation and Educational Development for the U.F. Clinical and Translational Science Institute. Methodologically, she conducts mixed-methods research studies employing both qualitative (interviews, mental models, and cultural consensus) and quantitative (experiments, content and textual analyses) approaches. Dr. Strekalova has been the principal investigator and co-investigator on numerous grants totaling over $1.5M in funding and published over 30 journal articles in a variety of healthcare, communication, and education journals.

**Juan S. Osorio-Valencia** is a biomedical engineer from Escuela de Inginería de Antioquia and Universidad Corporacion para Estudios en la Salud in Medellin, Colombia. He was named by Technology Review as one of the world’s top innovators under 35 for designing monitors to detect breathing problems in premature infants.

**Dr Rachael Parke** is an Associate Professor at the University of Auckland in the School of Nursing as well as the Nurse Senior Research Fellow in the Cardiothoracic and Vascular ICU at Auckland City Hospital. She has been involved in clinical research since 2004 and have been responsible for the CVICU becoming one of the busiest and most recognised intensive care research units in Australasia. Dr Parke has extensive involvement in many local and international investigator initiated clinical trials as an investigator and member of the trial steering committee and have an impressive publication and funding record. In addition to work in CVICU, she has been the Secretary of the Australian and New Zealand Intensive Care Society Clinical Trials Group (ANZICS-CTG), one of the world’s leading trials groups in critical care. The only non-physician to have held an elected office-bearer position within the ANZICS-CTG. Dr Parke also holds honorary positions with Monash University (Melbourne) and the Medical Research Institute of New Zealand (Wellington). She was awarded her PhD from the University of Auckland in 2014. Her thesis “High Flow Nasal Oxygen Therapy in Patients after Cardiac Surgery” was awarded the Vice-Chancellors Award - Best Doctoral Thesis 2014. Dr Parke has published 140 articles and been named on peer reviewed research grants exceeding NZ$9.6 million.

**Rogers Mwavu** is an Educator and an innovative software developer with a diverse skill-set and creative drive to software application development. Proficient at designing and formulating test automation frameworks, writing code in various object-oriented programming languages, feature development and implications. Rogers specializes in developing medical solutions that improve access to quality of health care services for underserved populations and promote quality of care.

**Nicole Martinez** received her JD from Harvard Law School and her doctorate in social sciences (comparative development/medical anthropology) from the University of Chicago. Her broader research interests concern the impact of new technologies on the treatment of vulnerable populations. Her graduate research included the study of cross-cultural approaches to mental health services in the Latine community and the use neuroscience in criminal cases. Her recent work in bioethics and neuroethics has focused on the ethics of AI and digital health technology, such as digital phenotyping or computer vision, for medical and behavioral applications. She has served as PI for research projects examining ethical issues regarding machine learning in health care, digital health technology, digital contact tracing, and digital phenotyping. She has examined policy and regulatory issues related to privacy and data governance, bias and oversight of machine learning and digital health technology. Her K01 career development grant, funded through NIMH, focuses on the ethics of machine learning and digital mental health technology. Recent research has included examining bias, equity and inclusion as it pertains to machine learning and digital health, as well as social implications of privacy and data protections on marginalized groups.

**Judy Wawira Gichoya** is Assistant Professor in the Department of Radiology and Imaging Sciences at Emory University School of Medicine. Dr. Gichoya is a multidisciplinary researcher, trained as both an informatician and an Interventional radiologist. Dr. Gichoya is a member of the Cancer Prevention and Control Research Program at Winship Cancer Institute. She holds professional memberships with Radiological Society of North America, American College of Radiology, Society of Interventional Radiology, Society of Imaging Informatics in Medicine and American Medical Informatics Association.

**Marzyeh Ghassemi** is an Assistant Professor at MIT in Electrical Engineering and Computer Science (EECS) and Institute for Medical Engineering & Science (IMES), and a Vector Institute faculty member holding a Canadian CIFAR AI Chair and Canada Research Chair. She holds MIT affiliations with the Jameel Clinic and CSAIL. Professor Ghassemi holds a Herman L. F. von Helmholtz Career Development Professorship, and was named a CIFAR Azrieli Global Scholar and one of MIT Tech Review’s 35 Innovators Under 35. Previously, she was a Visiting Researcher with Alphabet’s Verily. She is currently on leave from the University of Toronto Departments of Computer Science and Medicine. Prior to her PhD in Computer Science at MIT, she received an MSc. degree in biomedical engineering from Oxford University as a Marshall Scholar, and B.S. degrees in computer science and electrical engineering as a Goldwater Scholar at New Mexico State University.

**Dr Dina Demner-Fushman** leads research in information retrieval and natural language processing; providing clinical decision support through linking evidence (text and images) to patients’ data; answering clinical and consumer health questions; and extracting information from clinical text. Dr. Demner-Fushman earned her Doctor of Medicine degree from Kazan State Medical Institute in 1980, and clinical research Doctorate (PhD) in Medical Science degree from Moscow Medical and Stomatological Institute in 1989. She earned her M.S. and PhD in Computer Science from the University of Maryland, College Park in 2003 and 2006, respectively. She earned her B.A. in Computer Science from Hunter College, CUNY in 2000. Dr. Demner-Fushman is a lead investigator in several NLM projects in the areas of Information Extraction for Clinical Decision Support, EMR Database Research and Development, and Image and Text Indexing for Clinical Decision Support and Education. The outgrowths of these projects are the evidence-based decision support system in use at the NIH Clinical Center since 2009, an image retrieval engine, Open-i, launched in 2012, and an automatic customers’ requests answering service that supports NLM customer services since May 2014. She is the author of more than 160 articles and book chapters in the fields of information retrieval, natural language processing, and biomedical and clinical informatics. She has co-authored a textbook in Biomedical Natural Language Processing published in 2014.

**Dr Liam G McCoy** is a resident physician in Neurology at the University of Alberta, with broad-ranging research interests in the intersection between artificial intelligence and healthcare. He completed a Master of Science degree in Health Policy, Management and Evaluation with a focus on the ethical and equitable integration of A.I. in health systems contexts, including practica at the Vector Institute for Artificial Intelligence and the MIT Laboratory for Computational Physiology. He has worked to develop educational materials for A.I. in healthcare, and published regarding delineating the specific curricular needs for medical students in the A.I. realm. In addition, he has published extensively regarding issues of equity, fairness, and explainability in machine learning for healthcare, as well as regarding the interface between novel notions of personalized medicine and their forebears in the evidence based medicine movement.

**Dr Leo Anthony Celi** is a clinical research director and principal research scientist at the MIT Laboratory for Computational Physiology (LCP), he is also a practicing intensive care unit (ICU) physician at the Beth Israel Deaconess Medical Center (BIDMC). Dr Celi brings together clinicians and data scientists to support research using data routinely collected in the process of care. His group built and maintains the publicly-available Medical Information Mart for Intensive Care (MIMIC) database and the Philips-MIT eICU Collaborative Research Database, with more than 20,000 users from around the world. In addition, Leo is one of the course directors for HST.936 – global health informatics to improve quality of care, and HST.953 – collaborative data science in medicine, both at MIT. He is an editor of the textbook for each course, both released under an open access license. “Secondary Analysis of Electronic Health Records” has been downloaded more than a million times, and has been translated to Mandarin, Spanish, Korean and Portuguese. Dr Celi has spoken in more than 35 countries across 6 continents about the value of data and learning in health systems.

**Dr Robin L. Pierce** is Professor of A.I. and the Law at the University of Exeter. Her current research focuses on legal, policy, and ethical issues in the translation of AI-driven technologies in health, medicine, and the life sciences. This builds on a longstanding research line that brings together law, policy, and ethics to explore governance strategies for emerging technologies, e.g., genetics, synthetic biology, A.I., and neuroscience. Professor Pierce’s current research follows three main tracks examining regulatory approaches to addressing processes and impacts of technological innovation including- regulatory and policy approaches to translation of AI-driven and digital technologies for health and medicine, regulatory and governance approaches to addressing health inequities, participatory research, regulatory and policy approaches to the governance of AI-driven applications in the life sciences, and data governance. At Exeter, she is involved in several transdisciplinary collaborations and is a member of EGENIS, Microbes and Society, and is an affiliate member of the Wellcome Centre – Cultures and Environments for Health.
