# Supplementary material for: Peer review of GPT-4 technical report and systems card
Source: PLOS Digit Health. 2024 Jan 18;3(1):e0000417. doi: 10.1371/journal.pdig.0000417 (PMC10795998; doi:10.1371/journal.pdig.0000417)
Supplement: S3 File — (DOCX) [file pdig.0000417.s003.docx]

## **S3 File**

| Review criteria/prompt. | |
| --- | --- |
| **Methodology** | Is sufficient detail provided to understand the methodology used to produce their results? |
|  | Are the sources and size of information used to train and test the model clear and available for reproduction? |
|  | Has there been sufficient discussion on how data has been preprocessed? |
|  | Are there clear indications of the internal and external validation strategy? |
| **Findings** | Are the results presented in a transparent manner that best highlights the performance of the model? |
|  | Are there apparent omissions in this piece with respect to the overall goal of the project? |
|  | Is there sufficient evidence given to evaluate the model for algorithmic bias? |
|  | Is the model interrogated for the interpretability of outputs from the model? |
|  | Is there sufficient evidence addressing the security and privacy of the model? |
|  | Have areas of uncertainties or incompleteness been acknowledged? |
|  | Is there adequate exploration of the risks that may occur in the short and long term, and who they may impact? |
| **Recommendations** | Is sufficient evidence provided to support any conclusions and recommendations? |
|  | Is the context in which this model could be safely deployed adequately explored? |
|  | Are recommendations sufficiently specific to be actionable? |
|  | Are the authors qualified to comment on the areas they provide recommendations? |
|  | Are the values used to guide recommendations clear, and is it explicit who has made such judgments? |
|  | Does the manuscript maintain a tone of impartiality, consider alternative viewpoints, avoid advocacy, and treat sensitive issues with care? |
| **Other** | Does the document include the items suggested by its introduction, and, if necessary, does it explain what it does not cover? |
|  | What is the composition of team members involved in the project? |
|  | Is the document accessible to the general population or users using the data or supply information to train the model? |
